# Callous-unemotional Traits and Child Response to Teacher Rewards, Discipline, and Instructional Methods in Chinese Preschools: A Classroom Observation Study

**DOI:** 10.1007/s10802-023-01137-x

**Published:** 2023-10-17

**Authors:** Xinyi Cao, Matthew P. Somerville, Yiyun Shou, Zijing Xue, Jennifer L. Allen

**Affiliations:** 1 Department of Psychology and Human Development, Institute of Education, University College London, 25 Woburn Square, London, WC1H 0AA UK; 2 Lloyd’s Register Foundation Institute for the Public Understanding of Risk, National University of Singapore, Singapore, 117602 Singapore; 3 Saw Swee Hock School of Public Health, National University of Singapore and National University Health System, Singapore, 117549 Singapore; 4 Research School of Psychology, The Australian National University, Canberra, ACT 2600 Australia; 5 Early Childhood Education College, Shanghai Normal University, 100 Guilin Rd, Shanghai, 200234 China; 6 Department of Psychology, University of Bath, 10 West, Claverton Down, Bath, BA2 7AY UK

**Keywords:** Callous-unemotional traits, Teacher-child interaction, Rewards, Discipline, Instructional methods, School engagement

## Abstract

Atypical responses to teacher rewards, discipline and different forms of instructional methods have been identified as potential contributors to disruptive behavior, low school engagement, and academic underachievement in children with elevated callous-unemotional (CU) traits. To date, research on CU traits in schools has relied on interview or questionnaire methods and has predominantly been conducted in Western countries. Thus, the present study aims to investigate the relationships between CU traits and children’s responses to teacher rewards, discipline and instructional methods in the Chinese preschool context using classroom observation. Eight teachers (7 females, 1 male; M = 37.66 years) and 116 children (56% girls; M = 5.16 years) from two mainstream Chinese preschools participated in the study. Of the 116 eligible children, the behavior of 108 children from four classes were observed during classroom activities. Findings indicated that CU traits were not related to children’s responses to discipline, nor did CU traits moderate the relationship between instructional methods and children’s academic engagement. Higher CU traits predicted a greater frequency of one-to-one teacher-child interaction. Our findings offer initial insights into the potential of early school-based interventions in fostering engagement and prosocial behavior among children with CU traits. However, they also highlight the need for additional support for preschool teachers, who face the challenge of managing these high-risk children who appear to require more individual time and attention.

**Supplementary Information:**

The online version contains supplementary material available at 10.1007/s10802-023-01137-x.

Antisocial behavior brings substantial challenges to children’s success and well-being at school, including low engagement, school exclusion, school dropout, and problematic relationships with teachers and peers (Clark et al., 2002; Doumen et al., 2008; Miller & Olson, 2000). Children with antisocial behavior are a heterogeneous group, with different subtypes proposed to help better understand individual differences in the expression and severity of antisocial behavior, correlates, developmental trajectories, and response to intervention (Allen et al., 2020). Current theory highlighting temperamental pathways to antisocial behavior focuses on the presence of callous-unemotional (CU) traits, typified by low empathy, lack of guilt, shallow affect, and indifference to performance (Frick & Morris, 2004). CU traits are associated with childhood-onset antisocial behavior and can be reliably identified and assessed as early as the preschool years (Kimonis et al., 2016). Children with CU traits display more frequent, severe aggressive and disruptive behavior at school (Allen et al., 2018; Waschbusch et al., 2015). CU traits are associated with greater conflict and less closeness between teachers and students (Baroncelli & Ciucci, 2020), impaired peer relationships (Wagner et al., 2020), and academic underachievement (Horan et al., 2016). This has led to recent calls to recognize the potential utility of CU traits for addressing disruptive behavior in school, with teacher-child interaction identified as a potential target for intervention (Hwang et al., 2022; Willoughby et al., 2022).

Current theory emphasizes punishment insensitivity, an increased drive for social dominance, and reduced motivation for social affiliation as mechanisms impeding the development of conscience in children with CU traits (Pardini & Byrd, 2012; Waller & Wagner, 2019). School provides many opportunities for socialization, with teacher-child interaction viewed as an important means of supporting children to behave in a prosocial manner and to engage in learning (Aviles et al., 2006). Classroom management strategies draw on social-learning theory-based reward and discipline strategies to foster students’ engagement and reduce disruptive behavior (Webster-Stratton et al., 2001). However, low sensitivity to others’ distress, combined with a lack of guilt and interpersonal fearlessness may prevent the establishment of conditioned associations between affective discomfort and discipline (Blair, 1995; Waller & Wagner, 2019). Therefore, children with CU traits are more inclined to misbehave, and to repeat misbehavior following discipline (Byrd et al., 2014). Consistent with theory, qualitative research with high school teachers in England reported that students high in CU traits displayed uncaring and confrontational attitudes towards teachers when disciplined (Allen et al., 2016, 2018). Recent qualitative research in China found that preschool teachers perceived children with disruptive behavior and high CU traits as more likely to show uncaring or oppositional responses to discipline than children with disruptive behavior and low CU traits (Cao et al., 2023). Children with disruptive behavior and higher levels of CU traits did not appear guilty or concerned about the impact of their behavior on others, and refused to apologize following a transgression.

While punishment insensitivity is a well-established correlate of CU traits, findings for a link between CU traits and reward sensitivity are less consistent. The reasons for this are difficult to ascertain given study differences in sample characteristics, design, and methods (Blair & Zhang, 2020), but one explanation relates to the type of reward. Children with CU traits may respond more strongly to tangible rewards (e.g., money), and to rewards that enhance their social status or dominance (Pardini et al., 2003). Conversely, children with CU traits may be less responsive to affiliative rewards due to reduced motivation to seek others’ approval and to establish or maintain positive relationships (Viding & McCrory, 2019; Waller & Wagner, 2019). Qualitative interviews with high school teachers in England were mixed, as some teachers identified praise and a positive teacher-child relationship as increasing the prosocial behavior and school engagement of students high in CU traits, while others stated that neither social nor tangible rewards had any effect unless they could be used to enhance social status or achieve dominance over others (Allen et al., 2016, 2018). A recent qualitative study in China found more consistent views among preschool teachers, who reported that children with disruptive behavior high in CU traits were equally responsive to social rewards as children with disruptive behavior low in CU traits (Cao et al., 2023). Despite the value of classroom observation in providing an objective assessment of interactive exchanges between teachers and children in real-time, previous studies on CU traits and child responses to teacher discipline and rewards have relied on either student or teacher interview or questionnaire methods, which may be subject to mood, memory, and other subjective biases.

Only two previous studies have examined the link between CU traits and children’s responses to teacher classroom management strategies using a quantitative design. Consistent with theory, Baroncelli et al. (2022) found that peer-estimated insensitivity to teacher discipline was uniquely related to child-reported CU traits in a cross-sectional study of 695 Italian students (aged 11–15 years; 51% girls). Hwang et al. (2020) conducted a short-term longitudinal study during one school year with South Korean children (N = 218; aged 10–12 years, 52% boys). Child report of harsh teacher discipline predicted later decreased school engagement for children with lower, but not higher levels of CU traits, suggesting that CU traits may exert a protective effect against the deleterious effects of harsh teacher discipline on student engagement. Students were not responsive to teacher reward strategies regardless of their levels of CU traits, with increased rewards failing to predict greater school engagement. Hwang et al. (2020) attributed this finding to South Korea being a collectivist culture that prioritizes social obligations and success achieved through effort rather than innate talent. Such values might lead to greater engagement in South Korean children than in their Western peers, with external rewards playing a lesser role.

In addition to influencing children’s responses to classroom strategies, Horan et al. (2016) suggested that CU traits may elicit more frequent, harsh discipline from teachers, along with fewer rewards, leading to lower school engagement. Hwang et al. (2020) found that CU traits predicted decreased use of teacher rewards, suggesting that teachers found it difficult to maintain a warm and positive manner towards children with a callous-uncaring interpersonal style. Encouragingly, CU traits were not associated with harsh discipline at either data collection point. This is consistent with the results of a cross-sectional study of 138 first and second grade children in the United States where CU traits were unrelated to increased disciplinary infractions (Willoughby et al., 2022). Ciucci et al. (2014) found that CU traits were related to more teacher recorded formal warnings in 540 Italian children aged 11 to 14 years, however, in contrast to the other two studies, externalizing problems were not controlled for.

Teacher instructional methods also play an important role in promoting student academic engagement, motivation, and prosocial behavior. Traditional teacher-directed instructions are generally defined as emphasizing teachers’ dominance and control, and include the direct transmission of knowledge through lectures, demonstration and practice, and structured lessons with predetermined goals (Kikas et al., 2014). They have been criticized for potentially undermining students’ intrinsic motivation and engagement (Lerkkanen et al., 2012). However, teacher-directed instructions have been shown to be beneficial for children at risk, including those from disadvantaged social backgrounds (Adams & Carnine, 2003), with learning difficulties (Lovett et al., 2003), poor academic skills and poor task persistence, because teacher-directed instruction can strengthen academic skills from drill and practice and establish good work habits due to clear classroom rules and expectations (Kikas et al., 2014). Child-directed instructions are an alternative approach where children are viewed as active learners in constructing knowledge, and are encouraged to explore academic topics independently with teacher guidance and support (Lerkkanen et al., 2016). In child-directed classrooms, teachers value children’s needs and interests (McCombs, 2010), provide emotional support (Kikas & Tang, 2019), and encourage peer-cooperative learning (Henson, 2003). Child-directed instructions are associated with improved achievement, motivation, and socio-emotional and behavioral adjustment (Perry et al., 2007).

The poor academic performance of children high in CU traits has been attributed to their uncaring attitude towards academic failure, low intrinsic motivation, and reduced responsiveness to classroom management strategies (DeLisi et al., 2011; Horan et al., 2016). Another contributing factor may be that children with CU traits are less responsive to different instructional methods used by teachers to facilitate student achievement. For example, Bird et al. (2019) suggested that boys high in CU traits performed more poorly than girls in Science, due to their not receiving the same benefits from group work because of the lower empathy and poorer social competence that accompanies CU traits in boys compared to girls. In qualitative research, Cao et al. (2023) found that teachers perceived Chinese preschool children with disruptive behavior and CU traits as showing poorer engagement during teacher-directed activities but showed similar levels of engagement to their low-CU peers during one-to-one activities with teachers or individual learning activities that allowed them to select learning materials based on their interests. Furthermore, previous qualitative research indicated that teachers perceived high school children with CU traits as needing more intense monitoring and individual feedback in class to engage in academic work (Allen et al., 2018). A potential link between CU traits and children’s responses to different instructional methods has yet to be formally investigated using classroom observation methods.

The aim of the current study is to investigate the relationship between CU traits and the frequency of teacher use of rewards and discipline, as well as children’s responses to teacher rewards, discipline, and instructional methods. We focused on preschool children given that the early school years provide an important foundation for later academic achievement and career success (Melhuish, 2011). With one exception (Hwang et al., 2020), quantitative research examining CU traits and children’s responses to teacher rewards and discipline has focused on Western nations. The Chinese school context differs in its education system, including education policy and teacher training, and there are also known differences in the presentation and correlates of CU traits in East Asian and Western nations (Allen et al., 2021; Sng et al., 2020). The current study therefore used classroom observation to assess teacher-child interaction in real-time, in mainstream Chinese preschool classrooms. It was predicted that (1) CU traits would be related to significantly more frequent teacher discipline, and less frequent teacher rewards, as well as more negative child responses to discipline, less positive responses to affiliative rewards and more positive responses to tangible rewards. (2) The relationship between instructional methods and child academic engagement would be moderated by CU traits, such that CU traits would predict poorer engagement in teacher-directed activities, but better engagement in individual child-led learning activities. (3) CU traits would be significantly related to more frequent teacher use of one-to-one interaction. (4) CU traits would be significantly related to poor peer cooperation in group activities.

## Method

### Participants

Teachers and children were recruited from two public preschools in Shanghai, China. Eight teachers (7 female, 1 male) aged between 28 and 50 years old participated (M = 37.66 years; SD = 7.76). All teachers identified as Chinese, with teaching experience ranging from 6 to 29 years (*M* = 14.02 years, SD = 9.32). The number of students per classroom ranged from 26 to 38 children (*M* = 30, SD = 5.66). In Chinese preschools, typically there are two teachers in every class, therefore, each class register was halved and the two classroom teachers were given half of the list at random and asked to complete questionnaires for these students. Four out of 120 children attending the two schools were excluded because teachers identified them as having autism, developmental delay, or a significant health problem. The final sample therefore consisted of 116 children (52% girls, *n* = 62) aged between 4 and 6 years (M = 5.16 years, SD = 0.60) from four different classes (two classes in each preschool). All children were Chinese, and most were living with a two-parent family (*n* = 113, including 112 original two-parent families and a step/blended family), while the remainder belonged to a single-parent family (*n* = 3). The district where the two preschools were located has a higher GDP per capita than average in Shanghai (Xuhui District Local History Compilation Committee, 2021; Shanghai Municipal Bureau of Statistics, 2021).

### Measures

**Teacher and child demographic information.** Teachers provided information on their age, gender, ethnicity, and years of teaching experience. At the schools’ request, teachers, rather than parents, reported on child’s age, gender, ethnicity, and family type.

**CU Traits.** Teacher report of CU traits was obtained using the 24-item Inventory of Callous-Unemotional Traits (ICU; Frick, 2004). Teachers rated each item on a 4-point Likert scale from 0 ‘not at all true’ to 3 ‘definitely true’. The Mandarin translation of the ICU has good internal consistency for the total ICU score (*α* = 0.82) and is a valid measure in Chinese preschool children (Deng et al., 2016). Alpha for the total ICU score in the current sample was 0.87.

**Disruptive Behavior.** The Strengths and Difficulties Questionnaire (SDQ; Goodman, 1997) is a 25-item rating scale consisting of five subscales: conduct problems, hyperactivity, emotional symptoms, peer relationship problems and prosocial behavior. Teachers rate each item on a 3-point Likert scale from 0 ‘not at all true’ to 2 ‘certainly true’. The sum of the conduct problems and hyperactivity subscales was used to assess externalizing problems (Goodman et al., 2010). The validity of the Chinese version has been demonstrated by cross-scale correlations and its ability to discriminate between typically developing children and children with ADHD (Du et al., 2008). The alpha for SDQ externalizing problems was 0.72.

#### Classroom Observation of Children’s Response to Classroom Management Strategies and Instructional Methods

The Observed Child Engagement Scale (OCES; Rimm-Kaufman, 2005) and Social Development Lab-Kindergarten Coding System (SDL-K) (Rimm-Kaufman et al., 2007) were used to assess child behavior and academic engagement. The coding schemes were adapted to better fit the aims of the current study and the Chinese preschool context. We modified the original codes and included new codes generated from the literature on CU traits in the school setting (see Supplementary section for a detailed description of codes).^1^ The adjusted OCES assessed child engagement on the average score of four dimensions: engagement, self-reliance, attention, and disruptive behavior (reverse scored), with peer cooperation assessed as a separate dimension. Observers conducted classroom observation for a minimum of ten minutes, followed by five minutes of note taking and then assigned a rating from 1 ‘poor’ to 7 ‘good’ performance for each dimension (Ponitz et al., 2009). The OCES has shown good internal consistency (*α* = 0.91) and validity in preschool children (Rimm-Kaufman et al., 2009).

The adapted observation codes comprised ‘context’ and ‘frequency’ codes that were selected and modified from the SDL-K to enable us to address the current study aims. The adapted code list consisted of three main themes of codes: instructional methods, rewards and discipline. Instructional methods included 4 main types of instructions used by Chinese preschool teachers (Cao et al., 2023): teacher-directed activities, peer cooperation activities, individual learning activities and one-to-one teacher-child interaction. Unlike the first three instructional methods, which typically last for an entire observation window and do not usually occur simultaneously, one-to-one teacher-child interaction often only lasts for a few minutes and can occur alongside other classroom activities. For instance, a teacher might interact closely with children to support their learning during whole-class teaching or individual child-led learning activities. Accordingly, one-to-one teacher-child interactions were recorded as a frequency code, while other instructional methods were logged as context codes. We also recorded the frequency of children’s positive responses to teacher-child one-to-one instruction. Codes under the rewards and discipline themes are all frequency codes. The frequency of teachers’ use of rewards and discipline was recorded, along with children’s positive responses to teacher rewards and negative responses to discipline.

Teacher harsh discipline was recorded as a subcode under the broader code of discipline and comprised less than one-fifth of all disciplinary events during observation (*n* = 36, 17.22% of all disciplinary events). We also coded the frequency of tangible, social and activity rewards. Teachers predominantly used social rewards (*n* = 65, 83.33% of all rewards), with few occurrences of other types of rewards (*n* = 4, 5.13% for activity rewards and *n* = 9, 11.54% for tangible rewards). Therefore, in this study, only the total frequency of teacher rewards, and the frequency of children’s positive responses to social rewards will be reported.

Due to data collection taking place during the COVID-19 pandemic, observers were only permitted to stay in each classroom for one week. Therefore, we adapted the scheduling of the SDL-K to fit this observation period. Following adaptation, each target child was observed simultaneously by the two observers for a 15-minute period in total, with 10 min of observation using the context and frequency codes, followed by a five-minute additional period for completing the OCES ratings. Consequently, each child was observed only once, with their interactions with teachers being documented within the specific instructional method they were engaging in during the observation period. A pilot study was first conducted using videos of teacher-child interaction in Chinese preschool classrooms to ensure the feasibility and sensitivity of the adapted coding scheme, and to train the observers to an acceptable level of inter-rater reliability, pre-defined as kappa > 0.80 for categorical codes (e.g., type of instructional method), and ICCs > 0.75 for frequency codes (e.g., responses to rewards and discipline) and codes recorded on a rating scale (e.g., OCES codes) (Hallgren, 2012).

Schools and parents did not wish for classroom interactions to be filmed, therefore observational coding had to be conducted in real-time. During the pilot study, we found that a 15-minute window was sufficient for recording children’s academic engagement and one-to-one interactions with teachers. However, two challenges emerged. First, it was difficult to capture each child’s response when teachers rewarded or disciplined the whole class. Second, instances of teachers administering rewards or discipline to the target child were rare within this timeframe. We therefore recorded any instances of rewards or discipline clearly directed towards any specific child during the observation period, regardless of whether they were the target children for that specific observation window. This enabled us to collect data on rewards and discipline during a variety of instructional methods, to minimize the potential influence of instructional method type on the frequency of teacher rewards and discipline.

After a week-long observation in each classroom, we counted and collated the instances of each child receiving rewards and discipline, as well as their responses. Unlike rewards and discipline, which were recorded whenever they occurred throughout all classroom activities in one week, children’s academic engagement and one-to-one interactions with teachers were documented within the specific instructional activity they were participating in during the designated observation period. Inter-rater reliability analysis on 20% of the sample (*n* = 22) indicated strong agreement on the type of instructional method (kappa = 0.86). Similarly, observers were consistent for the remaining codes (frequency codes; continuous data), with intra-class correlation coefficients (ICCs) ranging from 0.76 to 1.

### Procedure

Once approval was obtained from the UCL Institute of Education research ethics committee, we asked for permission to approach teachers from each school’s principal. All teachers who were approached agreed to participate and provided written informed consent prior to data collection. Teachers then distributed information sheets and opt-out consent forms to caregivers. Initially, most parents refused to provide consent because they did not wish for their child to be filmed. Therefore, classroom observation was changed from video to live coding and revised information sheets and opt-out consent forms were sent out. No opt-out consent forms were returned following this new plan, and verbal assent was obtained from all children prior to the study.

This study took place in a one-month period from March to April 2021, during the middle of the spring term. Participating children were in their middle or final years of the three-year preschool period in the Chinese school system. This ensured that the teachers were well-acquainted with their students, thereby providing more valid and reliable information. Teachers completed the questionnaires within one week of receipt, prior to the classroom observation. Prior to formal academic activities, schools had an arrival session where children played freely in the classroom. Two trained observers arrived before this session and stayed in the classroom until school closure. To aid the identification of target children, we obtained rosters with children’s photos. This enabled the observers to associate each child’s face with their respective ID prior to formal observation. The observers also used the arrival sessions to familiarize themselves with the children, and this time also allowed the children to adjust to the observers’ presence. Importantly, on the first day of observation in each classroom, data collection did not commence until the observers were confident that the children had become accustomed to their presence, thus minimizing any undue influence on children’s behavior. Before each academic activity, the two observers randomly selected children in the class and agreed on the order of observation of the target children before observation commenced, to ensure that the observers could switch between children smoothly without interruption.

Once the target children were determined, the two observers placed themselves in a location enabling the simultaneous observation of the teachers’ and target children’s faces. Observers adjusted their position as necessary to sustain the visibility of both the teacher’s and target children’s faces. This was done while maintaining a sufficient distance from the teachers and children to prevent disturbing the class. The observers spent 15 min observing each target child, with a one-minute break between observation windows. The observation took place during classroom academic activities and stopped when children transitioned between activities, during snack time and when napping. At the end of each day, teachers were asked if this was (i) a typical day for their classroom, and (ii) a typical day for the children who had been observed. No special events were noted. The observers spent 4–5 days in each of the four classrooms in the two schools (*M* = 4.50 days; SD = 0.58).

### Data Analysis

Statistical analysis was performed using StataMP 17. All teachers completed the questionnaire packs. Observation data was missing for eight children who were absent from school. During the observation period, only a subset of children received a teacher’s reward, discipline, or engaged in one-to-one teacher-child interactions, and peer cooperation activities, with children who were observed but who did not experience these events coded as ‘not applicable (NA)’. This resulted in sample subgroups ranging from 39 to 116 children.

Before formal data analysis, we examined descriptive statistics for the main study variables (Table 1). To identify multicollinearity, interrelationships between variables, we ran two-tailed Pearson correlations between normally distributed continuous variables (e.g., CU traits and disruptive behavior), and Spearman correlations for correlation analysis involving count variables and non-normally distributed continuous variables (e.g., teacher use of discipline and child engagement). We conducted independent samples t-tests between normally distributed continuous variables and dichotomous variables (i.e., CU traits and gender) to test whether the main independent variables (CU traits and disruptive behavior) differed across demographic groups. All children in this study displayed positive responses every time they received one-to-one teacher-child interaction and social rewards. As a result, the relationships between proposed independent variables and child response to one-to-one teacher-child interaction and social rewards were not explored further. Furthermore, as the observed occurrences of tangible rewards were too infrequent to be analyzed (*n* = 9), our hypothesis that CU traits would be significantly related to more positive responses to tangible rewards was not tested either.

**Table 1:** Descriptive Statistics for the Main Study Variables

		N (children)	M (*SD*)	Range
CU traits		116	23.41 (10.54)	2–57
Disruptive behavior		116	5.90 (3.44)	0–16
Academic engagement	Teacher-directed activities	60	5.67 (1.33)	2–7
	Peer cooperation activities	39	6.21 (1.19)	1.25-7
	Individual learning activities	9	6.81 (0.35)	6–7
Peer cooperation		39	5.82 (1.73)	1–7
One-to-one teacher-child interaction		108	0.75 (1.14)	0–5
Positive responses to one-to-one teacher-child interaction		42	1.93 (1.05)	1–5
Teacher rewards		108	0.72 (1.06)	0–6
Positive responses to social rewards		44	1.48 (0.76)	1–4
Positive responses to tangible rewards		7	1.29 (0.49)	1–2
Harsh discipline		108	0.33 (0.79)	0–4
Teacher discipline		108	1.94 (3.35)	0–19
Negative responses to discipline		54	0.52 (1.24)	0–7

We constructed multivariate generalized linear models (GLMs) with cluster-robust standard errors to account for the fact that each teacher interacted with multiple children (McNeish et al., 2017). These models were used to investigate the relationships between CU traits and child negative responses to discipline, teacher use of rewards, discipline and harsh discipline, one-to-one teacher-child interaction, engagement, and peer cooperation. We included disruptive behavior and demographic variables as controlling variables in all models. In the models examining engagement and one-to-one teacher-child interaction, we also accounted for instructional methods as engagement and one-to-one teacher-child interaction were recorded within the specific instructional method that occurred during each child’s set observation period, rather than across different instructional methods as for rewards and discipline.

The choice of distribution for the multi-level GLMs depended on the nature of the dependent variable. GLMs with a negative binomial distribution were used to examine relationships between CU traits and the frequency of teacher rewards, discipline and one-to-one teacher-child interaction as these three dependent variables were count variables and were over-dispersed (Walters, 2007). GLMs with binomial regression and logit link (i.e., logistic regression) were used to investigate whether CU traits and disruptive behavior influenced the likelihood of a child being recorded as NA in receiving total rewards, social rewards, total discipline, and one-to-one teacher-child interaction. Logistic regression was also applied to model the proportion of instances of harsh discipline (event) to total instances of discipline (trial), as well as children’s negative responses (event) to discipline (trial). GLMs with a normal distribution (i.e., multi-level linear regression) were used to explore how CU traits and instructional methods influenced child engagement and peer cooperation. We also explored whether instructional methods moderated the effect of CU traits on children’s engagement by testing the interaction between CU traits and instructional method.^2^

## Results

### Preliminary Analyses

Table 1 presents descriptive statistics for the main study variables. Bivariate correlations (Table 2) indicated that CU traits were significantly related to more severe disruptive behavior and poorer engagement. Disruptive behavior was related to younger child age, more frequent total discipline, harsh discipline and poorer engagement. Poor engagement was significantly related to more frequent teacher discipline, child negative response to discipline and poorer peer cooperation. Child negative response to discipline was also significantly related to more frequent teacher discipline and harsh discipline. Male gender was significantly related to more frequent teacher rewards and discipline. Teacher harsh discipline was significantly related to more frequent discipline and more one-to-one teacher-child interaction. No other significant relationships were identified.

**Table 2:** Bivariate Correlations for the Demographics and Main Study Variables

	1	2	3	4	5	6	7	8	9	10
1. Age (*n* = 116)										
2. Gender (*n* = 116)	− 0.03									
3. CU traits (*n* = 116)	− 0.003	0.03								
4. Disruptive behavior (*n* = 116)	− 0.20 *	− 0.08	0.55**							
5. Rewards Total (*n* = 108)	− 0.14	0.27**	− 0.08	0.002						
6. Discipline Total (*n* = 108)	− 0.12	0.29**	0.17	0.31**	0.14					
7. Harsh discipline (*n* = 108)	− 0.14	0.17	0.13	0.23*	0.19	0.60**				
8. One-to-one Interaction (*n* = 108)	− 0.15	0.01	0.06	− 0.03	0.18	0.16	0.19*			
9. Engagement (n = 108)	− 0.12	− 0.07	− 0.22*	− 0.26**	0.08	− 0.24*	− 0.15	0.05		
10. Peer cooperation (*n* = 39)	− 0.08	− 0.12	− 0.04	− 0.20	0.20	− 0.004	0.13	0.16	0.48**	
11. Negative responses to discipline (*n* = 54)	− 0.06	0.11	0.11	0.25	0.05	0.44**	0.50**	0.06	− 0.30*	− 0.13

Note. ** *p* < .01 * *p* < .05, Pearson/point-biserial correlation coefficient and Spearman correlation coefficient were reported

#### CU Traits, Rewards and Discipline

Our multivariate logistic analysis (Table 3) indicated a significant relationship between child gender and the likelihood of receiving total rewards (*n* = 108), social rewards (*n* = 108), discipline (*n* = 108), and negative responses to teacher discipline (*n* = 54) while controlling for disruptive behavior and demographic variables. Boys were more likely to receive total rewards, social rewards and discipline, and were more likely to respond negatively to discipline. The multivariate negative binomial regressions (Table 4) showed that gender was significantly related to the frequency of teacher rewards and discipline after accounting for other variables, with boys receiving more frequent rewards and discipline than girls. Older children tended to receive less frequent rewards than younger ones. No other significant relationships were found between CU traits and outcomes, including teacher use of reward, teacher use of social rewards, teacher total or harsh discipline, and child negative response to discipline.

**Table 3:** Logistic Model Results for Reward, Discipline and One-to-One Teacher-Child Interaction

		B	RSE (B)	Z	*p*	Odds Ratio
	Reward (*n* = 108)					
Child age		− 0.46	0.24	-1.94	0.053	0.63
Child gender		1.06	0.13	8.06	< 0.001	2.90
CU traits		− 0.02	0.03	− 0.65	0.514	0.98
Disruptive behavior		0.01	0.05	0.24	0.810	1.01
	Social rewards (*n* = 108)					
Child age		− 0.46	0.25	-1.88	0.060	0.64
Child gender		0.88	0.14	6.14	< 0.001	2.40
CU traits		− 0.02	0.03	− 0.79	0.431	0.98
Disruptive behavior		0.05	0.05	0.92	0.356	1.05
	Discipline (*n* = 108)					
Child age		− 0.30	0.49	− 0.61	0.544	0.74
Child gender		1.29	0.43	3.02	0.003	3.63
CU traits		0.03	0.03	1.19	0.235	1.03
Disruptive behavior		0.06	0.07	0.85	0.393	1.06
	Harsh discipline (*n* = 54)					
Child age		0.17	0.26	0.64	0.524	1.19
Child gender		0.23	0.37	0.61	0.539	1.26
CU traits		− 0.01	0.02	− 0.27	0.786	0.99
Disruptive behavior		− 0.03	0.05	− 0.63	0.530	0.97
	Negative responses to discipline (*n* = 54)					
Child age		− 0.02	0.25	− 0.06	0.949	0.98
Child gender		1.10	0.28	3.93	< 0.001	3.00
CU traits		0.03	0.03	1.25	0.212	1.03
Disruptive behavior		0.04	0.09	0.41	0.679	1.04
	One-to-one teacher-child interaction occurrence (*n* = 108)					
Child age		0.51	0.37	1.38	0.169	1.66
Child gender		− 0.36	0.39	− 0.91	0.360	0.70
CU traits		− 0.02	0.02	-1.08	0.281	0.98
Disruptive behavior		0.07	0.05	1.42	0.155	1.07
Instructional method 1		0.35	0.50	0.69	0.493	1.41
Instructional method 2		− 0.57	0.86	− 0.66	0.510	0.57

Note: Male gender, the occurrence of rewards, social reward, discipline, and one-to-one teacher-child interaction = 1

Instructional method 1 = peer cooperation activities, Instructional method 2 = individual learning activities

The reference category for instructional methods is teacher-directed activities. RSE = robust standard error

**Table 4:** Negative Binomial Model Results for Teacher Use of Rewards, Discipline and One-to-One Teacher-Child Interaction

		B	RSE (B)	Z	*p*	95%CI
	Reward (*n* = 108)					
Child age		− 0.40	0.19	-2.09	0.037	[-0.78, − 0.03]
Child gender		0.83	0.17	4.79	< 0.001	[0.49, 1.17]
CU traits		− 0.01	0.02	− 0.92	0.358	[-0.05, 0.02]
Disruptive behavior		0.001	0.03	0.02	0.982	[-0.07, 0.07]
	Discipline (*n* = 108)					
Child age		− 0.37	0.29	-1.31	0.191	[-0.93, 0.19]
Child gender		0.74	0.30	2.48	0.013	[0.15, 1.33]
CU traits		0.01	0.03	0.42	0.672	[-0.04, 0.07]
Disruptive behavior		0.10	0.07	1.45	0.147	[-0.04, 0.24]
	One-to-one teacher-child interaction (*n* = 108)					
Child age		− 0.56	0.30	-1.85	0.065	[-1.15, 0.03]
Child gender		− 0.09	0.34	− 0.27	0.786	[-0.75, 0.57]
CU traits		0.04	0.02	2.25	0.025	[0.005, 0.07]
Disruptive behavior		− 0.10	0.05	-2.05	0.040	[-0.18, − 0.004]
Instructional method 1		− 0.40	0.34	-1.17	0.243	[-1.06, 0.27]
Instructional method 2		0.10	0.67	0.14	0.885	[-1.22, 1.42]

Note: Male gender = 1, Instructional method 1 = peer cooperation activities, Instructional method 2 = individual learning activities

The reference category for instructional methods is teacher-directed activities. RSE = robust standard error. CI = Confidence Interval

#### CU Traits and Instructional Methods

The multivariate linear regression model results (Table 5) indicated that child age, disruptive behavior and instructional methods were significantly related to academic engagement while controlling for other variables. Children with younger age and more severe disruptive behavior displayed poorer academic engagement. Children had better engagement in peer cooperation and individual learning activities than children in teacher-directed activities. There was no significant relationship between CU traits and academic engagement (*n* = 108) or peer cooperation when controlling for other variables (*n* = 39). To test whether CU traits moderated the effect of instructional methods on child engagement, we compared a model regressing child engagement on CU traits, disruptive behavior, instructional methods and demographics, and a model with the addition of the interaction effect between CU traits and instructional methods. The addition of the interaction term did not significantly improve model fit, suggesting that CU traits did not significantly moderate the effect of instructional methods on engagement, *F*(2, 7) = 1.46, *p* = .295. The multivariate negative binomial regressions (Table 4) showed that CU traits and disruptive behavior were significantly related to one-to-one teacher-child interaction, with CU traits associated with more frequent interactions and disruptive behavior associated with less frequent interactions. No other predictors significantly influenced the likelihood of a child receiving one-to-one teacher-child interactions.

**Table 5:** Linear Regression Model Results for Child Academic Engagement and Peer Cooperation

		B	RSE (B)	*t*	*p*	95%CI
	Child academic engagement (*n* = 108)					
Child age		− 0.43	0.15	-2.82	0.026	[-0.79, − 0.07]
Child gender		− 0.31	0.23	-1.38	0.210	[-0.84, 0.22]
CU traits		− 0.01	0.02	− 0.90	0.399	[-0.05, 0.02]
Disruptive behavior		− 0.06	0.02	-2.65	0.033	[-0.11, − 0.01]
Instructional method 1		0.59	0.22	2.64	0.033	[0.06, 1.12]
Instructional method 2		1.02	0.13	7.86	< 0.001	[0.71, 1.32]
	Peer cooperation (*n* = 39)					
Child age		− 0.69	0.42	-1.67	0.140	[-1.68, 0.29]
Child gender		− 0.08	0.55	− 0.15	0.888	[-1.38, 1.22]
CU traits		0.01	0.02	0.55	0.597	[-0.04, 0.06]
Disruptive behavior		− 0.10	0.07	-1.47	0.186	[-0.26, 0.06]

Note: Male gender = 1, Instructional method 1 = peer cooperation activities, Instructional method 2 = individual learning activities,

The reference category for instructional methods is teacher-directed activities. RSE = robust standard error. CI = Confidence Interval

Instructional method was not controlled for peer cooperation as peer cooperation can only be observed during peer cooperation activities

## Discussion

We investigated CU traits, teacher-child interaction (teacher rewards, discipline and instructional methods), and children’s peer cooperation and academic engagement using classroom observation. Our hypothesis that CU traits would be significantly associated with more frequent teacher discipline was not supported, aligning with previous studies in South Korea and the United States that found no significant relationship between CU traits and the frequency or severity of teacher discipline (Hwang et al., 2020; Willoughby et al., 2022). Hwang et al. suggested that, unlike the findings on CU traits and parental discipline (e.g., Waller et al., 2017), teachers may not use harsh discipline towards children high in CU traits due to their professional ethics, legal responsibilities, and training. Consistent with this explanation, disruptive behavior was also unrelated to more frequent discipline or harsh discipline in the current study. Our study extends the understanding of the relationship between CU traits and teacher use of discipline to Chinese preschool children, as well as to a wide spectrum of observed discipline strategies ranging from mild to severe disciplinary practices (Cao et al., 2023).

Contrary to our hypothesis, teachers in the current study implemented reward strategies at a similar frequency to all children regardless of their level of CU traits. Similar to the findings for discipline, it may be that teacher training and professional ethics ensured that teachers maintained the use of reward-based strategies with children whose behavior is difficult to manage. However, this result contrasts with Hwang et al.’s (2020) findings that CU traits were related to reduced teacher rewards at the start of the school year as well as 9 months later for South Korean children. This may be because Hwang et al. relied on child retrospective report of teacher rewards, which may have been influenced by mood, memory or other biases such as poor-quality teacher-child relationships, known to be related to CU traits (Horan et al., 2016). As such, children with high CU traits may perceive teachers’ behavior towards them as less positive than it appears to independent observers. The inconsistent findings may also be due to the younger age of children in the current study compared to the Hwang et al. sample (10–12 years) given that the severity of CU traits and disruptive behavior increases with child age (Kemp et al., 2019). Thus, it may be that it is easier for Chinese preschool teachers to maintain the use of reward-based strategies given the milder expression of CU traits and associated disruptive behavior in early childhood. However, it should be noted that our findings may also be attributed to social desirability bias. Despite the observers’ efforts to minimize their presence, which included maintaining distance from teachers and students, avoiding any reactions to teacher behavior (e.g., smiling, nodding or frowning), and arriving before arrival sessions and conducting a week-long observation period to allow teachers to acclimate to the observers’ presence, teachers may have used more positive strategies than usual due to a desire to demonstrate their professional competence.

Our hypothesis that CU traits would be related to reduced sensitivity to teacher discipline was not supported. This contrasts with theory and previous research findings that teacher and child self-reported insensitivity to discipline was related to CU traits when accounting for antisocial behavior (e.g., Allen et al., 2018; Hwang, Allen, Kokosi, & Bird, 2021). These inconsistent findings may be due to the use of classroom observation in the current study in comparison to questionnaires or teacher interviews. Another possible explanation is that the preschool-aged children in the current study were more amenable to discipline than children in past studies attending elementary school or high school due to the greater malleability of child temperament in early childhood. Consistent with this explanation, parent training interventions comprising discipline and reward-based strategies delivered in early childhood achieve better outcomes for children with elevated CU traits compared to interventions delivered in middle-to-late childhood (Hawes et al., 2014). Furthermore, the positive attention of adults (parents, teachers) may exert a stronger motivational influence on behavior in early childhood than in adolescence, when young people shift to seeking the approval of peers (Laursen & Veenstra, 2021). Children usually have different teachers for different subjects and therefore spend less time with a single teacher in high school than in earlier periods of schooling. Thus, teachers in preschools have more opportunities to establish a close relationship with children high in CU traits, which may then facilitate the effectiveness of teacher discipline and reward strategies (Allen et al., 2018).

Our hypothesis that CU traits would be significantly related to decreased sensitivity to rewards that are social or affiliative in nature was not tested due to the lack of variability in responses to this form of reward. One possible explanation for the lack of variation could be that rewards were equally effective for all children regardless of their level of CU traits, consistent with the findings of a recent qualitative interview study with Chinese preschool teachers (Cao et al., 2023). In past mixed methods studies in English high school students (Allen et al., 2016, 2018), quantitative results showed no relationships between CU traits and teacher or child self-reported reward sensitivity, while qualitative findings indicated that responsivity to rewards was reduced or even problematic (e.g., rewards used to boast to others or abuse of privileges). Cao et al. suggested that the more optimistic picture for young school children in China may be due to the higher value placed on rewards from teachers than children from Western nations due to the emphasis on respect for elders in Confucianism (Chen & Chung, 1994), the greater malleability of temperament in early childhood, or the greater power of adult positive attention in early childhood compared to adolescence (Allen et al., 2016).

However, the lack of variability in responses to social rewards may also be due to the sensitivity of coding scheme. We divided child responses into simple positive and negative categories, which may not have captured nuanced individual differences in reward sensitivity. Future research could benefit from developing a multi-tiered coding scheme to capture a broader spectrum of responses. Moreover, the small sample size for social rewards (*n* = 44) limited our ability to observe a diverse range of child responses. Nevertheless, these preliminary findings can serve as a foundation to inform the design of future studies featuring larger samples. Our prediction on child responses to tangible rewards was not tested either due to the few occurrences in the current study (*n* = 9). This finding may reflect the trend in professional teacher training for social rewards over tangible rewards in China (Sun, 2008; Yu, 2018) due in part to concerns around the potential negative impact of tangible rewards on children’s intrinsic motivation (Warneken & Tomasello, 2008).

In line with our hypothesis, we found that CU traits were significantly related to more frequent one-to-one teacher-child interaction, accounting for disruptive behavior and demographic variables. Our results provide preliminary evidence for a unique role of CU traits in shaping one-to-one teacher-child interaction, and align with previous qualitative research indicating that high school teachers viewed children with CU traits as needing close supervision (Allen et al., 2018). The current study extends this work through the use of classroom observation with Chinese preschool children, with findings highlighting the need for greater support for teachers to effectively promote the classroom engagement of these high-risk children.

Similar to past research highlighting the role of punishment insensitivity in explaining the link between CU traits and low academic achievement (Hwang et al., 2021), our results showed that poorer academic engagement was related to higher CU traits and children’s negative responses to discipline. However, the significance of CU traits as a predictor disappeared after controlling for disruptive behavior, instructional methods, and other demographic variables. Results showed that instructional methods were significantly related to child academic engagement even after accounting for other variables, with teacher-directed activities being by far the least engaging form of instructional method for children, followed by peer cooperation and individual learning activities. These findings are consistent with past studies indicating that children display better engagement when teaching practices are more child-directed than teacher-directed (Lerkkanen et al., 2016; Perry et al., 2007), and when teachers are able to focus on supporting one individual child (McWilliam et al., 2003).

Our prediction that CU traits would be related to different levels of engagement across different instructional formats was not supported. This may be due to the young age of children in the current study. Unlike the high school setting, preschool children are often closely supervised, and as such, teachers can prevent problematic behavior or intervene early to ensure that children are engaged and cooperative. Another possibility is that due to covid-related restrictions on the length of the observation period, only nine children participated in individual learning activities, limiting our ability to detect significant effects. Therefore, further research employing a longer observation period or with video recording is needed to replicate current findings. Similar to social rewards, there was a lack of variability in children’s positive responses to one-to-one teacher-child interactions. This could be due to either no impact of CU traits on the effectiveness of one-to-one interaction, limitations in the sensitivity of the coding scheme, or to constraints related to sample size.

Contrary to our prediction and Bird et al. (2019)’s suggestion that impairments in empathy and social competence may prevent children with elevated CU traits from performing well in peer learning activities, our results indicated no significant relationship between CU traits and peer cooperation in learning activities. One possible reason for this unexpected result could be the ample materials, physical space and monitoring provided by teachers in peer cooperative learning activities in the present study. As a result, this may have reduced the likelihood of common sources of conflict between children during peer activities, such as competition for toys. It is also possible that the more covert disruptive behavior of children high in CU traits was not identified by observers, leading to inflated ratings of their engagement in peer cooperative activities. Our findings suggest that one-to-one teacher attention and child-directed instruction may be helpful in promoting academic engagement in Chinese preschool children regardless of their level of CU traits or disruptive behavior. However, similarly to other observational variables, the small sample of children engaging in peer cooperation activities may undermine the strength of our findings.

The interpretation of the current findings needs to be considered in light of study limitations. The most salient limitation is that we had limited instances of variables assessing teacher-child interaction that could be measured for every child participant. We may have been able to record more instances of the target behaviors and activities if the study had been conducted over a longer period. However, due to the strict entry policy during COVID-19 and the requirement for live coding, data collection was limited to one week in each classroom and each child could only be observed once. Within this time frame, we may not have captured enough desired interactive exchanges between teachers and children to be able to detect small but significant relationships between variables. Furthermore, while we were able to assess rewards and discipline across various instructional methods, the number of children in the observed activities related to academic engagement and one-to-one interactions was not evenly balanced, despite our efforts to randomly select children for observation during these specific activities. Although we controlled for instructional methods when exploring academic engagement and one-to-one instruction, the uneven distribution of observed children across various academic activities may still introduce potential bias. However, the aim of this research was to explore teacher-child interaction in real classroom settings. We did not manipulate or alter the classroom routines, and as such, the uneven distribution of observed children across various academic activities is reflective of the natural rhythm of classroom schedules.

Past qualitative research by Cao et al. (2023) in Chinese preschools suggested that teachers perceive some children as always being well behaved (and thus discipline is not needed), while others are perceived as rarely behaving well, and are therefore rarely rewarded. In addition, teachers identified teacher-directed activities as the most commonly used instructional methods, compared to peer cooperation and individual learning. A longer observation period therefore may not significantly increase the likelihood of receiving teacher rewards or discipline for some children or obtain similar numbers of different academic activities, but future research is needed to determine the optimal length of the observation period to capture the desired teacher behaviors in the Chinese preschool context. English high school teachers reported that the disruptive behavior of children with CU traits may be more common in the school hallways and playground due to an awareness of the reduced likelihood of detection (Allen et al., 2016). Thus, another possible approach to obtain sufficient instances of the target behaviors could be through behavior observation in the hallway and playground settings.

It should also be noted that there were limitations of using cluster-robust standard errors for the current data. This method typically requires a large sample size and number of clusters, and our study sample was relatively small with only 8 clusters. In addition, the assumption that observations between clusters (teacher) should be unrelated may not hold for some of our dependent variables. After calculating ICCs for a third level (classroom), we found that for teacher-child one-to-one interaction, teacher rewards, and peer cooperation, effects of individual teachers from the same classroom could be correlated. However, due to the even smaller number of clusters for classroom (4 clusters only), we proceeded with cluster-robust SE adjusted for teacher level clusters. Therefore, results should be interpreted with caution and future research should feature a larger sample size to enable more comprehensive modeling approaches. Finally, this study had a cross-sectional design, and thus cannot determine the direction of relationships between CU traits and teacher frequency of rewards and discipline, or child responses to rewards, discipline, and instructional methods. All children were Chinese and attended mainstream schools in economically developed areas, thus, findings may not generalize to children from other nations or from a socially disadvantaged background. Our findings therefore need to be replicated in a study featuring more schools to include children with a greater severity of CU traits and disruptive behavior.

Our findings provide preliminary evidence that young children may similarly be responsive to teacher reward and discipline strategies and different forms of instructional methods regardless of their level of CU traits. This suggests that teachers can focus on implementing strategies that are effective for all children, including reward-based strategies and child-directed activities, rather than needing to adopt a personalized approach. Our results paint somewhat a more optimistic picture for the responsiveness of children with CU traits in Chinese preschools to rewards, discipline, and instructional methods than previous research with children in the elementary and high school periods (e.g., Allen et al., 2016; Allen et al., 2018; Hwang et al., 2020). Therefore, current study findings suggest the potential importance of early identification and intervention in schools. However, our findings need to be interpreted cautiously due to study limitations and the preliminary nature of this research. Future research should employ longitudinal investigation using classroom observation methods over different periods of schooling to enable more comprehensive information to be obtained about the relationship between CU traits, classroom management strategies, and instructional formats.

### Electronic Supplementary Material

Below is the link to the electronic supplementary material.

Supplementary Material 1

## Footnotes

1 *The manual and form of our coding schemes are available upon request to the corresponding author.*

2 The interaction effect between CU traits and different types of instructional methods was not tested for peer cooperation as peer cooperation can only be measured during peer cooperation activities.
