# Supplementary material for: Callous-unemotional Traits and Child Response to Teacher Rewards, Discipline, and Instructional Methods in Chinese Preschools: A Classroom Observation Study
Source: Res Child Adolesc Psychopathol. 2023 Oct 17;52(3):339–52. doi: 10.1007/s10802-023-01137-x (PMC10896772; doi:10.1007/s10802-023-01137-x)
Supplement: Supplementary file 1 — Supplementary Material 1 [file 10802_2023_1137_MOESM1_ESM.docx]

**Supplementary Section: Definitions for Observation Codes**

This Supplementary Section provides definitions for the observation codes used in our study. To better fit our research aims, these codes were adapted from The Observed Child Engagement Scale (OCES; Rimm-Kaufman, 2005) and Social Development Lab-Kindergarten Coding System (SDL-K) (Rimm-Kaufman et al., 2007), as well as derived from literature on CU traits in the school setting. For the complete observation protocol with a more detailed description of the codes, please contact the corresponding author.

**Revised OCES codes:**

**Engagement:**

The engagement dimension evaluates a child’s active involvement in tasks and instructions, reflecting both intensity and persistence. Highly engaged children are frequently involved in opportunities offered by teachers, showing marked enthusiasm for, and concentration on tasks. Conversely, children with low engagement scores may exhibit aimless wandering in class, engage in activities that do not fit with the teachers’ agenda or sit at their desks without engaging in any activities.

**Self-reliance:**

The self-reliance dimension assesses the extent to which a child displays autonomy, responsibility, and personal initiative. Self-reliant children take control of their own learning, show resilience in the face of challenges, seek adult assistance only after using their own resources, and take the lead in activities or problem-solving with peers. Children low in self-reliance may tend to follow rather than lead. They may lack confidence, exhibit passivity, seek help for even minor challenges before attempting tasks, and await instructions before initiating any actions.

**Attention:**

The attention dimension captures the degree to which children exhibit sustained, focused, and direct attention to ongoing classroom activities. Children with high levels of attention consistently concentrate on classroom tasks and show little or no distraction in response to other classroom events. In contrast, inattentive children are easily distracted or engaged in activities other than planned classroom activities (e.g., fidgeting, playing with their clothes), and need frequent prompting and redirecting from teachers to maintain focus.

**Disruptive behavior:**

The disruptive behavior dimension evaluates overall child disruptiveness in the classroom. Children with high disruptive behavior scores may violate classroom rules or frequently interrupt classroom proceedings, such as calling out inappropriately, annoying others and chatting to peers when it is not allowed. Children with low disruptive behavior scores are generally compliant, cooperative and adhere to classroom rules. These children may be inattentive at times, but they remain quiet and do not disturb others in the classroom.

**Peer cooperation:**

The peer cooperation dimension evaluates the degree to which children positively engage with others during peer cooperative learning activities. Children with high scores on peer cooperation show pronounced sociability and enjoyment in peer interaction. These children adeptly cooperate and negotiate with their peers to complete group tasks. Conversely, children scoring low on this dimension may display negative engagement with their peers, including conflict, domineering behavior, and drawing inappropriate attention to themselves.

**Revised SDL-K codes:**

**Teacher-directed activity (context code):**

Teacher-directed activities refer to instructions where the teacher decides on the content, pacing, and structure of learning, such as skill-and-drill practices. These activities can be conducted in a whole-class format, led by one teacher, or in smaller groups guided by multiple teachers.

**Peer cooperation activity (context code):**

Peer cooperation activities, such as role-playing, group tasks, and peer sharing/discussion, are child-directed nature and emphasize on peer collaboration in either small groups or pairs. In these activities, teachers take on a more supportive role, providing guidance, materials, and space, rather than leading the activity directly. The structure of these activities can vary; at times, teachers allocate children into groups or pairs with assigned tasks. Alternatively, in a more flexible format, children are given the autonomy to choose tasks based on their own interests and select their group members or partners for pair work.

**Individual learning activity (context code):**

Individual learning activities are highly children-directed. These activities allow children the freedom to choose their learning materials and engage in self-directed learning and personal exploration. Children in individual learning activities may choose a book from the classroom library and read it at their own pace, work on individual art projects or played alone with different materials in exploration centers in the classroom.

**One-to-one teacher-child interaction (frequency code):**

One-to-one teacher-child interaction involves a teacher providing close supervision and personalized guidance to an individual child. For example, during a teacher-directed activity, a teacher might approach a child who is having difficulty with learning task and provide tailored instruction. In a peer cooperation activity, if a child is socially withdrawn, the teacher might engage in a one-on-one conversation to encourage participation. During individual learning activities, such as individual reading, a teacher might discuss the pictures in the book with the child to enhance their comprehension and stimulate their imagination. To avoid overlap with reward and discipline codes, instances where teachers give rewards or discipline to individual children are not considered or coded as one-to-one teacher-child interaction in the current research.

**Positive response to one-to-one teacher-child interaction (frequency code):**

A positive response to one-to-one teacher-child interaction is characterized by active engagement in the interaction. This can be demonstrated through adherence to teacher instructions, responsiveness to the teacher's prompts or questions, and an evident interest in the activity or topic at hand. Positive emotional responses, such as smiling, can also be indicative of a positive response to the interaction.

**Reward (frequency code):**

This code refers to the use of positive reinforcement to encourage desirable behaviors. Rewards can be tangible, such as stickers, or social, such as praise and hugs. They can also be activity rewards, such as extra play time or being the teacher’s helper. Each reward should be coded with its specific type.

**Discipline (frequency code):**

This code refers to strategy used to correct or manage inappropriate or disruptive behaviors. This can include strategies such as disciplinary cues (e.g., “you have to stop talking”), move person (e.g., timeout), move object (e.g., the teacher takes away the box of crayons that the child was breaking.), loss of privileges and criticism.

**Harsh discipline (frequency codes):**

Harsh discipline is a subcode under the code “discipline”. It refers to overly punitive discipline that can negatively impact a child's self-esteem and emotional well-being. This includes actions such as yelling, belittling, using a stern and intimidating tone of voice, or making harsh criticism about the child rather than their behavior. For instance, a teacher might express disappointment in a way that shames or embarrasses the child, such as saying ‘Stop crying like a baby!’

**Positive response to rewards (frequency code):**

Children expressing enjoyment and appearing motivated by rewards were coded as positive responses to rewards. This could be demonstrated, for instance, by a child displaying a cheerful demeanor upon receiving a reward, showing increased interest in the task at hand, or improving their performance or will complete classroom tasks in anticipation of a reward.

**Negative response to discipline (frequency code):**

Children who do not comply with behavioral requests, or who display indifferent or aggressive attitudes towards discipline, are coded as exhibiting negative responses. For instance, a child might ignore a teacher's request, continue engaging in disallowed activities, or refuse to carry out actions as instructed by the teacher. Negative responses can also manifest as crying, verbal aggression, or other forms of disruptive behavior.
